# Supplementary material for: Effect of Cannabidiol on Human Peripheral Blood Mononuclear Cells and CD4+ T Cells
Source: Int J Mol Sci. 2023 Oct 4;24(19):14880. doi: 10.3390/ijms241914880 (PMC10573927; doi:10.3390/ijms241914880)
Supplement: Supplementary file 1 [file ijms-24-14880-s001.zip › Furgiuele et al_Suppl Fig 1.pdf]

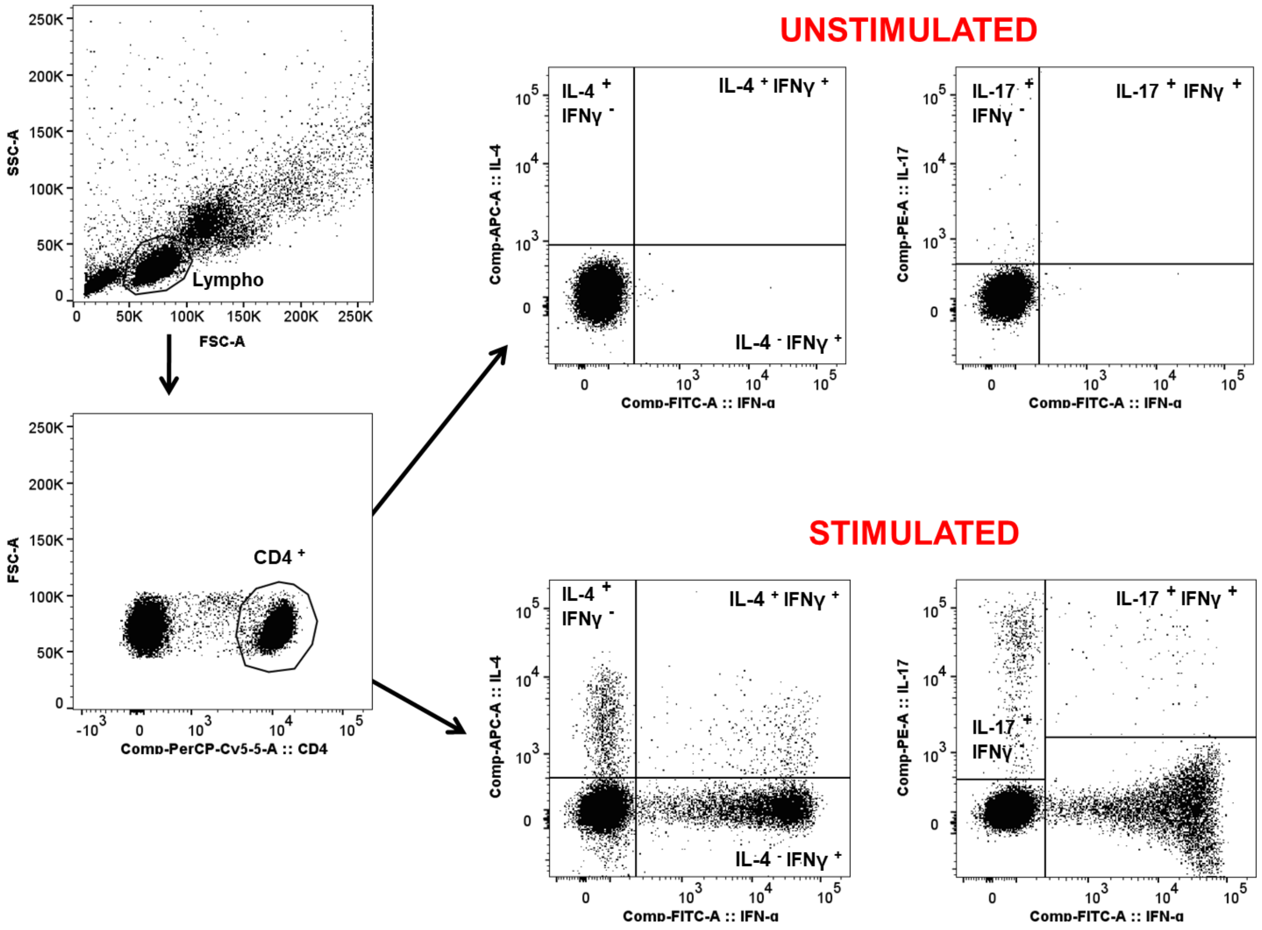

Figure S1. Gating strategy for 3-color intracellular cytokine staining (ICS).

Total lymphocytes (Lympho) are identified using a morphological dot plot (FSC vs SSC). Lymphocytes are then visualized on a bi-parametric dot plot FSC vs CD4 and a gate is established around CD4 positive cells (CD4+). Finally, CD4 positive cells are gated on two bi-parametric dot plots IL-4 vs IFN-γ and IL-17 vs IFN-γ to select four different subpopulations: IL-4$^-$/IFN-γ$^+$ (Th1) and IL-4$^+$/IFN-γ$^-$ (Th2) cells on the IL-4 vs IFN-γ plot; IL-17$^+$/IFN-γ$^-$(Th17) and IL-17$^+$/IFN-γ$^+$ (Th1-Th17) cells on the IL-17 vs IFN-γ plot.
