# Supplementary material for: Effect of Cannabidiol on Human Peripheral Blood Mononuclear Cells and CD4+ T Cells
Source: Int J Mol Sci. 2023 Oct 4;24(19):14880. doi: 10.3390/ijms241914880 (PMC10573927; doi:10.3390/ijms241914880)
Supplement: Supplementary file 1 [file ijms-24-14880-s001.zip › Furgiuele et al_Suppl Table 1.pdf]

Table S1
Intracellular staining antibodies for cytokine measurement in CD4+ positive cells

Antibodies and supplier for the flow cytofluorimetric assay of intracellular cytokine staining. Further details are reported in the method section.

Target	Supplier	Species	Clone; Isotype	Conjugation	Volume (µL/test)	Reference
Human CD4	Biolegend-Campoverde, Italy	mouse	SK3; IgG1, k	PerCPCy5.5	2.5	344608
Human IFN-γ	Biolegend-Campoverde, Italy	mouse	B27; IgG1, k	FITC	2.5	506504
Human IL-4	Biolegend-Campoverde, Italy	rat	MP4-25D2; IgG1, k	APC	2.5	500812
Human IL-17	Becton Dickinson, Italy	mouse	N49-653; IgG1, k	PE	10	560486
