# Supplementary material for: Effect of Cannabidiol on Human Peripheral Blood Mononuclear Cells and CD4+ T Cells
Source: Int J Mol Sci. 2023 Oct 4;24(19):14880. doi: 10.3390/ijms241914880 (PMC10573927; doi:10.3390/ijms241914880)
Supplement: Supplementary file 1 [file ijms-24-14880-s001.zip › Furgiuele et al_Suppl Table 2.pdf]

Table S2: Real-Time PCR probes for gene expression

Gene Symbol	UniGene ID	Interrogated Sequence *RefSeq/GenBank mRNA*	Detected Coding Transcripts	Amplicon Context Sequence	Chromosome Location	Amplicon Lenght	Annealing temperature (°C)	Efficiency (%)
**TNF-alpha**	Hs.241570	NC_000006.11, NG_007462.1, NG_012010.1, NT_007592.15, NT_113891.2, NT_167244.1, NT_167245.1, NT_167246.1, NT_167247.1, NT_167248.1, NT_167249.1	ENST00000328965, ENST00000445232, ENST00000594551, ENST00000443707, ENST00000412275, ENST00000449264, ENST00000577810, ENST00000326294, ENST00000448781, ENST00000420425, ENST00000394126, ENST00000356271, ENST00000394128, ENST00000394127, ENST00000422942, ENST00000501516, ENST00000536318, ENST00000431269, ENST00000376122, ENST00000383496, ENST00000264203, ENST00000375144, ENST00000375142, ENST00000401084, ENST00000439554	GGGGTCTTCCAGCTGGAGAAGGGTGACCGACTCAGCGCTGAGATCAATCGGCCCGACTATCTCGACTTTGCCGAGTCTGGGCAGGTCTACTTTGGGATCATTGCCCT GTGAGGAGGACGAACATC	6:31545204-31545328	95	60	99
**IL-17**	Hs.41724	NC_000006.11, NT_007592.15	ENST00000340057	CCCGGACTGTGATGGTCAACCTGAACATCCATAACCGGAATACCAATACCAATCCCAAAAGGTCCTCAGATTACTACAACCGATCCACCTCACCTTGGAATCTCCACCGCAATGAGGACCCTGAGAGATATCCC	6:52052498-52053880	104	60	99
**IFN-gamma**	Hs.856	NC_000012.11, NG_015840.1, NT_029419.12	ENST00000229135	CACAACCCATGGGATCTTGCTTAGGTTGGCTGCCTAGTTGGCCCCTGAGATAAAGCCTTGTAATCACATAGCCTTGCCTAATTAGTCAGAAAACAAAGGATTAAGTGAGACAGTCACAGGATATAGGA	12:68548835-68548962	98	60	99

Gene Symbol	UniGene ID	Interrogated Sequence *RefSeq/GenBank mRNA*	Detected Coding Transcripts	Amplicon Context Sequence	Chromosome Location	Amplicon Lenght	Annealing temperature (°C)	Efficiency (%)
**RPS18**	Hs.627414	NC_000006.11, NT_007592.15, NT_113891.2, NT_167245.1, NT_167247.1, NT_167248.1, NT_167249.1	ENST00000454021, ENST00000486781, ENST00000484321, ENST00000211372, ENST00000477055, ENST00000476288, ENST00000439602, ENST00000474973, ENST00000457341, ENST00000494232, ENST00000434122	GTGGAACGTGTGATCACCATTATGCA GAATCCACGCCAGTACAAGATCCCAG ACTGGTTCTTGAACAGACAGAAGGAT GTAAAGGATGGAAAATACA	6:33243742-33243838	67	60	98
**FOXP3**	Hs.247700	NC_000023.10, NG_021311.1, NT_079573.4, NG_007392.1	ENST00000376207, ENST00000557224, ENST00000518685, ENST00000376197, ENST00000376199, ENST00000455775	GAAGGCAAACATGCGTGTGAACCAGT GGTAGATCTCATTGAGTGTCCGCTGC TTCTCTGGAGCCTCCAGGATGGCCCA GCGGATGAGCGTGGCGTAGGTGAAA GGGGGTCGCATGTTGTGGAACTTGAA GTAGTCCATGTTGTGGAGGAACTCTG GGAAT	X:49108152-49110385	129	60	102
**GATA3**	Hs.524134	NC_000010.10, NG_015859.1, NT_008705.16	ENST00000379328, ENST00000346208	TGCAAAGGAGCTCACTGTGGTGTCTG TGTTCCAACCACTGAATCTGGACCCC ATCTGTGAATAAGCCATTCTGACTCAT ATCCCCTATTTAACAGGGTC	10:8116131-8116229	69	60	101
**NURR1**	Hs.563344	NC_000002.11, NG_011821.1, NT_005403.17	ENST00000339562, ENST00000426264, ENST00000409572, ENST00000409108, ENST00000429376, ENST00000424077, ENST00000421709, ENST00000539077	TAGTAAACCGACCCGGAGTGCGGCAT CATCTCCTCAGACTGGGGGGGCAGGT GGCTGTGTTGCTGGTAGTTGTGCATC TGAATGTCTTCTACCTTAA	2:157186334-157186430	67	60	99
**RORC**	Hs.256022	NC_000001.10, NT_004487.19	ENST00000356728, ENST00000318247, ENST00000392697	GGAGGTGCTGGAAGATCTGCAGCCTT TCCACATGCTGGCTACACAGGCTCCG AAGCTTCCCCTTGGGTGGCAGCTTTG CCAGGATGCTTTGGCGATGAGTCTTG CAGAGATGATGATGAAAGGCCAGCTC CAGATTGTACTGCAGCTGTTCTA	1:151780037-151783880	123	60	100

Gene Symbol	UniGene ID	Interrogated Sequence *RefSeq/GenBank mRNA*	Detected Coding Transcripts	Amplicon Context Sequence	Chromosome Location	Amplicon Lenght	Annealing temperature (°C)	Efficiency (%)
**STAT1**	Hs.731486	NC_000002.11, NG_008294.1, NT_005403.17	ENST00000361099, ENST00000409465, ENST00000392322, ENST00000392323, ENST00000424722, ENST00000454414, ENST00000432058, ENST00000540176	CCAGTCTTGCTTTTCTAACCACTGTGC CAGGTACTGTCTGATTTCCATGGGAAA ACTGTCATCATAAAGCTGGTGAACCTG CTCCAGGAATTTTGAGTCAAGCTGCT GAAGTTCGTACCACTGAGACATCCTG CCACCTTG	2:191873833-191878261	111	60	97
**STAT3**	Hs.463059	NC_000017.10, NT_010783.15, NG_007370.1	ENST00000264657, ENST00000404395, ENST00000389272	GGTGTCACACAGATAAACTTGGTCTTC AGGTATGGGGCAGCGCTACCTGGGTC AGCTTCAGGATGCTCCTGGCTCTCTG GCCGACAATACTTTCCGAATGCCTCCT CCTTGGGAATGTCAGGATAGAGATAG ACCAGTGGAGACACCAGGATATTGGT	17:40469200-40474414	125	60	96
**STAT4**	Hs.80642	NC_000002.11, NG_012852.1, NT_005403.17	ENST00000358470, ENST00000392320, ENST00000413064, ENST00000409995, ENST00000450994	AGTTTTGAAGAAGAATCGTTGCCATGG TTTCATTGTTAGAAGCTGCCTCCCAGT CTTGATTTTCAATCCATTGGGCCAACA GATGCCGAATTTCCATGGGAAAGTTG TCAT	2:192011434-192012862	81	60	94
**STAT6**	Hs.524518	NC_000012.11, NT_029419.12, NG_021272.1	ENSG00000166888	ATAGACACATGTTCTATGTGGTCATGC AACTAAGGTGCCAGCTATACATTTAAC ATATCCTAGGTACATACACGTTCACAC AGCTATACACGAAGAATCTCAGCCCTT GTACTTTTGCATAGTCTCATACACGTA TCAGAAGCCTCCACC	12:57489602-57489751	120	60	101
**TBET**	Hs.272409	NC_000017.10, NG_012166.1, NT_010783.15	ENST00000177694	GTTTTATAACTATTTTCCCAACTGAGC AGATGACATGATGAAAGGAACAGAAA CAGTGTTATTAGGTTGGAGGACACCG ACTAATTTGGGAAACGGATGAAG	17:45822708-45822809	72	60	97
